# The non-centrosymmetric polymorph of (quinolin-8-ol-κ^2^ *N*,*O*)(quinolin-8-olato-κ^2^ *N*,*O*)silver(I)

**DOI:** 10.1107/S160053681300281X

**Published:** 2013-02-02

**Authors:** Zhen-Bin Jia, Yi Zhao, Qiu-Jia Wen, Ai-Qing Ma

**Affiliations:** a School of Pharmacy, Guangdong Medical College, Dongguan 523808, People’s Republic of China; b School of Clinical Medicine, Guangdong Medical College, Dongguan 523808, People’s Republic of China

## Abstract

The title compound, [Ag(C_9_H_6_NO)(C_9_H_7_NO)], crystallizes as a non-centrosymmetric polymorph. The structure was previously reported by Wu *et al.* [(2006). *Acta Cryst.* E**62**, m281–m282] in the centrosymmetric space group *Pbcn*. The Ag^I^ ion displays a distorted tetra­hedral coordination geometry defined by two N and two O atoms from a neutral quinolin-8-ol ligand (HQ) and a deprotonated quinolin-8-olate anion (Q^−^). The dihedral angle between the two ligands is 47.0 (1)°. Strong O—H⋯O hydrogen bonds link the mol­ecules into a supra­molecular chain along the *a*-axis direction.

## Related literature

For the centrosymmetric polymorph, see: Wu *et al.* (2006 ▶).
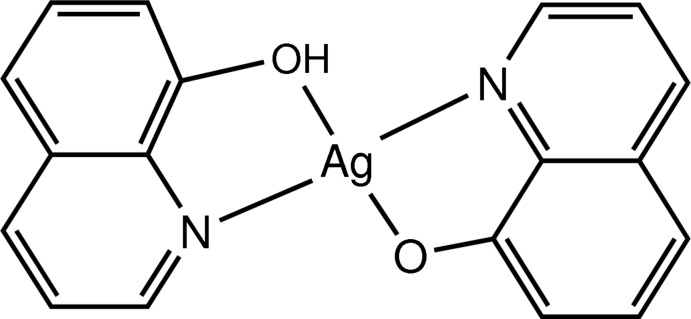


## Experimental

### 

#### Crystal data

[Ag(C_9_H_6_NO)(C_9_H_7_NO)]
*M* *_r_* = 397.17
Orthorhombic, 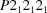

*a* = 7.2320 (3) Å
*b* = 10.4857 (6) Å
*c* = 18.9398 (10) Å
*V* = 1436.25 (13) Å^3^
*Z* = 4
Mo *K*α radiation
μ = 1.42 mm^−1^
*T* = 293 K
0.19 × 0.18 × 0.15 mm

#### Data collection

Bruker SMART diffractometer
Absorption correction: multi-scan (*SADABS*; Bruker, 2002 ▶) *T*_min_ = 0.884, *T*_max_ = 1.000
8103 measured reflections
2554 independent reflections
2305 reflections with *I* > 2σ(*I*)
*R*_int_ = 0.033

#### Refinement

*R*[*F* ^2^ > 2σ(*F* ^2^)] = 0.027
*wR*(*F* ^2^) = 0.057
*S* = 1.08
2554 reflections
208 parameters
H-atom parameters constrained
Δρ_max_ = 0.41 e Å^−3^
Δρ_min_ = −0.34 e Å^−3^
Absolute structure: Flack (1983 ▶), 1056 Friedel pairs
Flack parameter: −0.02 (3)

### 

Data collection: *SMART* (Bruker, 2002 ▶); cell refinement: *SAINT* (Bruker, 2002 ▶); data reduction: *SAINT*; program(s) used to solve structure: *SHELXS97* (Sheldrick, 2008 ▶); program(s) used to refine structure: *SHELXL97* (Sheldrick, 2008 ▶); molecular graphics: *ORTEP-3 for Windows* (Farrugia, 2012 ▶); software used to prepare material for publication: *WinGX* (Farrugia, 2012 ▶).

## Supplementary Material

Crystal structure: contains datablock(s) I, global. DOI: 10.1107/S160053681300281X/hg5282sup1.cif

Structure factors: contains datablock(s) I. DOI: 10.1107/S160053681300281X/hg5282Isup2.hkl

Additional supplementary materials:  crystallographic information; 3D view; checkCIF report

## Figures and Tables

**Table 1:** Hydrogen-bond geometry (Å, °)

*D*—H⋯*A*	*D*—H	H⋯*A*	*D*⋯*A*	*D*—H⋯*A*
O2—H2*A*⋯O1^i^	0.82	1.73	2.495 (3)	154

Symmetry code: (i) 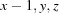.

**Fig. 1.**
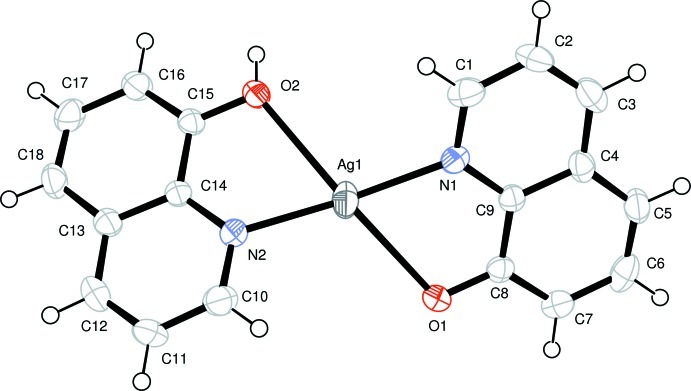
The molecular structure of (I).

**Fig. 2.**
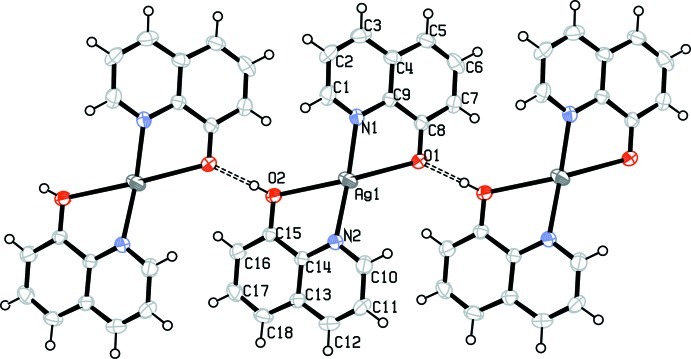
View of the hydrogen-bonding chain of (1). Hydrogen bonds are drawn as dashed lines.

**Fig. 3.**
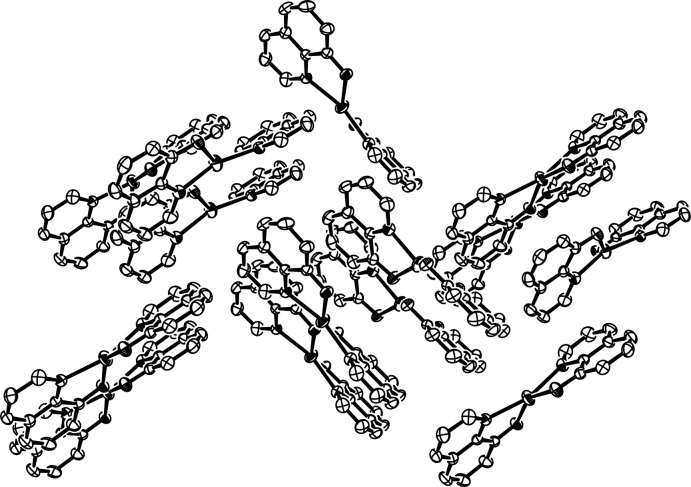
View of the packing. H atoms have been omitted for clarity.

## supplementary crystallographic information

### Comment

In the title compound,(I), the Ag ion is four-coordinated by two nitrogen atoms and two oxygen atoms from two 8-hydroxyquinoline ligands, forming a distorted tetrahedral geometry (Fig. 1). The two 8-hydroxyquinoline ligands are different in their mode of the coordination. One is a neutral ligand while the other is deprotonated. The dihedral angle between two 8-hydroxyquinoline mean planes is 47.0 (1)°. A polymorph (II) of the structure has been previously reported by Wu *et al.* (2006) in the centrosymmetric space group *Pbcn* with *a* = 11.434 (2)Å, *b* = 14.817 (3)Å, *c* = 8.7828 (18)Å. The Ag-N bondlengths of 2.174 (3), 2.176 (3)Å in (I) are shorter than the value of 2.2377 (19)Å for (II) while the Ag-O bondlengths of 2.596 (2), 2.649 (2)Å are longer than the value of 2.4831 (17)Å found for (II). Inter-molecular O_H···O hydrogen bonding between HQ and Q^-^ ligands form a supramolecular chain structure (Table 1, Fig. 2). Weak π-π interactions are observed between neighboring aromatic rings [dihedral angle 2.0 (2)°] with the centroid-to-centroid distance of 3.75 (1) Å, which is favorable to increase the stability of the structure (Fig. 3).

### Experimental

A methanol solution (15 ml) of 8-hydroxyquinoline(HQ) (0.075 g,0.5 mmol) was mixed with an aqueous solution (5 ml) of AgNO_3_ (0.085 g, 0.5 mmol). Ammonia solution was dropped into the mixture under stirring until it was almost clear. Then it was filtered. Yellow single crystals, suitable for X-ray, were obtained after several days.

### Refinement

The H atoms on C atoms and O atom were placed in idealized positions and refined as riding atoms with C—H = 0.93 Å and O—H = 0.84 (2) Å, with *U*_iso_(H) = 1.2*U*_eq_(C).

### Crystal data

**Table:**

[Ag(C_9_H_6_NO)(C_9_H_7_NO)]	*F*(000) = 792
*M**_r_* = 397.17	*D*_x_ = 1.837 Mg m^−^^3^
Orthorhombic, *P*2_1_2_1_2_1_	Mo *K*α radiation, λ = 0.71073 Å
Hall symbol: P 2ac 2ab	Cell parameters from 3403 reflections
*a* = 7.2320 (3) Å	θ = 2.8–29.6°
*b* = 10.4857 (6) Å	µ = 1.42 mm^−^^1^
*c* = 18.9398 (10) Å	*T* = 293 K
*V* = 1436.25 (13) Å^3^	Block, yellow
*Z* = 4	0.19 × 0.18 × 0.15 mm

### Data collection

**Table:**

Bruker SMART diffractometer	2554 independent reflections
Radiation source: Enhance (Mo) X-ray Source	2305 reflections with *I* > 2σ(*I*)
Graphite monochromator	*R*_int_ = 0.033
Detector resolution: 10.3592 pixels mm^-1^	θ_max_ = 25.1°, θ_min_ = 2.9°
ω scans	*h* = −8→7
Absorption correction: multi-scan (*SADABS*; Bruker, 2002)	*k* = −9→12
*T*_min_ = 0.884, *T*_max_ = 1.000	*l* = −22→21
8103 measured reflections	

### Refinement

**Table:**

Refinement on *F*^2^	Secondary atom site location: difference Fourier map
Least-squares matrix: full	Hydrogen site location: inferred from neighbouring sites
*R*[*F*^2^ > 2σ(*F*^2^)] = 0.027	H-atom parameters constrained
*wR*(*F*^2^) = 0.057	*w* = 1/[σ^2^(*F*_o_^2^) + (0.022*P*)^2^ + 0.3181*P*] where *P* = (*F*_o_^2^ + 2*F*_c_^2^)/3
*S* = 1.08	(Δ/σ)_max_ = 0.001
2554 reflections	Δρ_max_ = 0.41 e Å^−^^3^
208 parameters	Δρ_min_ = −0.34 e Å^−^^3^
0 restraints	Absolute structure: Flack (1983), 1056 Friedel pairs
Primary atom site location: structure-invariant direct methods	Flack parameter: −0.02 (3)

### Special details

**Table:**

Geometry. All e.s.d.'s (except the e.s.d. in the dihedral angle between two l.s. planes) are estimated using the full covariance matrix. The cell e.s.d.'s are taken into account individually in the estimation of e.s.d.'s in distances, angles and torsion angles; correlations between e.s.d.'s in cell parameters are only used when they are defined by crystal symmetry. An approximate (isotropic) treatment of cell e.s.d.'s is used for estimating e.s.d.'s involving l.s. planes.
Refinement. Refinement of *F*^2^ against ALL reflections. The weighted *R*-factor *wR* and goodness of fit *S* are based on *F*^2^, conventional *R*-factors *R* are based on *F*, with *F* set to zero for negative *F*^2^. The threshold expression of *F*^2^ > σ(*F*^2^) is used only for calculating *R*-factors(gt) *etc*. and is not relevant to the choice of reflections for refinement. *R*-factors based on *F*^2^ are statistically about twice as large as those based on *F*, and *R*- factors based on ALL data will be even larger.

### Fractional atomic coordinates and isotropic or equivalent isotropic displacement parameters (Å^2^)

**Table:**

	*x*	*y*	*z*	*U*_iso_*/*U*_eq_	
C17	0.3321 (5)	0.5388 (4)	0.2207 (2)	0.0428 (11)	
H17	0.2247	0.4904	0.2240	0.051*	
C18	0.4685 (6)	0.5214 (4)	0.2684 (2)	0.0413 (11)	
H18	0.4528	0.4635	0.3051	0.050*	
Ag1	0.87935 (4)	0.85088 (3)	0.101833 (16)	0.04331 (11)	
C6	1.4013 (5)	1.0168 (4)	−0.0955 (2)	0.0428 (10)	
H6	1.5083	1.0187	−0.1227	0.051*	
O2	0.5261 (3)	0.7849 (2)	0.10618 (14)	0.0319 (6)	
H2A	0.4318	0.7863	0.0821	0.048*	
O1	1.2184 (3)	0.8451 (3)	0.05683 (12)	0.0347 (6)	
C8	1.2323 (5)	0.9235 (4)	0.00265 (19)	0.0279 (8)	
C7	1.3899 (6)	0.9315 (4)	−0.03871 (19)	0.0360 (9)	
H7	1.4903	0.8791	−0.0286	0.043*	
C9	1.0833 (4)	1.0077 (3)	−0.01372 (18)	0.0264 (8)	
C13	0.6343 (5)	0.5908 (3)	0.26259 (18)	0.0300 (8)	
C15	0.5067 (5)	0.6993 (3)	0.15794 (19)	0.0268 (8)	
N1	0.9246 (4)	1.0033 (3)	0.02576 (16)	0.0309 (8)	
N2	0.8161 (4)	0.7483 (3)	0.19863 (16)	0.0280 (7)	
C11	0.9365 (5)	0.6477 (5)	0.3027 (2)	0.0406 (10)	
H11	1.0325	0.6407	0.3352	0.049*	
C16	0.3498 (5)	0.6289 (4)	0.1662 (2)	0.0372 (9)	
H16	0.2520	0.6408	0.1350	0.045*	
C14	0.6564 (4)	0.6808 (3)	0.20672 (18)	0.0245 (8)	
C2	0.7975 (5)	1.1738 (4)	−0.0422 (2)	0.0435 (11)	
H2	0.6996	1.2297	−0.0497	0.052*	
C4	1.0976 (5)	1.0959 (3)	−0.07134 (19)	0.0325 (9)	
C3	0.9487 (6)	1.1781 (4)	−0.0839 (2)	0.0411 (11)	
H3	0.9540	1.2359	−0.1211	0.049*	
C10	0.9497 (5)	0.7328 (4)	0.2455 (2)	0.0404 (10)	
H10	1.0574	0.7804	0.2404	0.048*	
C1	0.7905 (5)	1.0843 (4)	0.0121 (2)	0.0414 (11)	
H1	0.6851	1.0818	0.0402	0.050*	
C12	0.7824 (6)	0.5764 (4)	0.3098 (2)	0.0388 (10)	
H12	0.7742	0.5172	0.3462	0.047*	
C5	1.2597 (5)	1.0971 (4)	−0.1118 (2)	0.0401 (10)	
H5	1.2705	1.1526	−0.1498	0.048*	

### Atomic displacement parameters (Å^2^)

**Table:**

	*U*^11^	*U*^22^	*U*^33^	*U*^12^	*U*^13^	*U*^23^
C17	0.033 (2)	0.037 (3)	0.059 (3)	−0.0098 (19)	0.0030 (19)	0.007 (2)
C18	0.047 (2)	0.035 (3)	0.042 (3)	0.002 (2)	0.010 (2)	0.015 (2)
Ag1	0.04359 (16)	0.04830 (19)	0.03805 (17)	−0.00869 (17)	0.00683 (17)	0.01115 (17)
C6	0.039 (2)	0.054 (3)	0.036 (2)	−0.013 (2)	0.013 (2)	−0.006 (2)
O2	0.0300 (11)	0.0322 (14)	0.0337 (14)	−0.0030 (11)	−0.0046 (13)	0.0085 (14)
O1	0.0289 (12)	0.0401 (16)	0.0350 (14)	0.0029 (14)	−0.0021 (11)	0.0112 (15)
C8	0.0259 (18)	0.031 (2)	0.027 (2)	−0.0041 (18)	−0.0037 (16)	−0.0017 (18)
C7	0.0288 (18)	0.041 (2)	0.038 (2)	0.002 (2)	−0.002 (2)	−0.0019 (18)
C9	0.0271 (19)	0.027 (2)	0.0249 (19)	−0.0052 (17)	−0.0029 (15)	−0.0009 (15)
C13	0.0324 (18)	0.029 (2)	0.028 (2)	0.008 (2)	0.0037 (18)	0.0008 (15)
C15	0.0291 (18)	0.023 (2)	0.028 (2)	0.0052 (16)	0.0018 (16)	0.0009 (17)
N1	0.0246 (16)	0.0364 (19)	0.0318 (18)	−0.0006 (15)	0.0009 (12)	0.0021 (14)
N2	0.0268 (15)	0.0289 (18)	0.0283 (17)	0.0006 (14)	0.0010 (13)	0.0005 (15)
C11	0.037 (2)	0.049 (3)	0.036 (2)	0.005 (2)	−0.0110 (16)	0.006 (2)
C16	0.034 (2)	0.035 (2)	0.043 (2)	−0.004 (2)	−0.0063 (17)	0.0065 (18)
C14	0.0257 (17)	0.022 (2)	0.0257 (18)	0.0052 (16)	0.0037 (14)	−0.0016 (15)
C2	0.041 (2)	0.042 (3)	0.048 (3)	0.014 (2)	−0.009 (2)	0.007 (2)
C4	0.039 (2)	0.029 (2)	0.029 (2)	−0.008 (2)	−0.0038 (17)	0.0036 (15)
C3	0.053 (2)	0.030 (2)	0.040 (3)	−0.0032 (19)	−0.0094 (19)	0.0092 (19)
C10	0.032 (2)	0.050 (3)	0.040 (2)	0.002 (2)	−0.0016 (18)	0.000 (2)
C1	0.031 (2)	0.049 (3)	0.044 (3)	0.008 (2)	0.0000 (19)	−0.003 (2)
C12	0.051 (2)	0.034 (3)	0.030 (2)	0.003 (2)	0.0009 (19)	0.0078 (19)
C5	0.044 (2)	0.041 (3)	0.035 (2)	−0.010 (2)	0.001 (2)	0.011 (2)

### Geometric parameters (Å, º)

**Table:**

C17—C18	1.349 (6)	C13—C14	1.427 (5)
C17—C16	1.406 (5)	C15—C16	1.362 (5)
C17—H17	0.9300	C15—C14	1.436 (5)
C18—C13	1.407 (6)	N1—C1	1.316 (5)
C18—H18	0.9300	N2—C10	1.322 (5)
Ag1—N2	2.174 (3)	N2—C14	1.364 (4)
Ag1—N1	2.176 (3)	C11—C12	1.348 (5)
Ag1—O1	2.596 (2)	C11—C10	1.408 (6)
Ag1—O2	2.649 (2)	C11—H11	0.9300
C6—C5	1.361 (6)	C16—H16	0.9300
C6—C7	1.401 (5)	C2—C3	1.349 (5)
C6—H6	0.9300	C2—C1	1.394 (6)
O2—C15	1.336 (4)	C2—H2	0.9300
O2—H2A	0.8200	C4—C3	1.400 (5)
O1—C8	1.319 (4)	C4—C5	1.400 (6)
C8—C7	1.386 (5)	C3—H3	0.9300
C8—C9	1.427 (5)	C10—H10	0.9300
C7—H7	0.9300	C1—H1	0.9300
C9—N1	1.370 (4)	C12—H12	0.9300
C9—C4	1.434 (5)	C5—H5	0.9300
C13—C12	1.403 (5)		
			
C18—C17—C16	121.1 (4)	C9—N1—Ag1	120.8 (2)
C18—C17—H17	119.5	C10—N2—C14	118.7 (3)
C16—C17—H17	119.5	C10—N2—Ag1	118.3 (2)
C17—C18—C13	120.1 (4)	C14—N2—Ag1	122.0 (2)
C17—C18—H18	120.0	C12—C11—C10	118.9 (3)
C13—C18—H18	120.0	C12—C11—H11	120.5
N2—Ag1—N1	162.38 (12)	C10—C11—H11	120.5
N2—Ag1—O1	117.62 (9)	C15—C16—C17	121.6 (3)
N1—Ag1—O1	70.00 (10)	C15—C16—H16	119.2
N2—Ag1—O2	69.00 (9)	C17—C16—H16	119.2
N1—Ag1—O2	110.96 (9)	N2—C14—C13	121.4 (3)
O1—Ag1—O2	156.21 (9)	N2—C14—C15	119.7 (3)
C5—C6—C7	121.7 (4)	C13—C14—C15	118.8 (3)
C5—C6—H6	119.2	C3—C2—C1	118.9 (4)
C7—C6—H6	119.2	C3—C2—H2	120.5
C15—O2—H2A	109.5	C1—C2—H2	120.5
C8—O1—Ag1	108.2 (2)	C3—C4—C5	123.0 (4)
O1—C8—C7	122.7 (3)	C3—C4—C9	118.1 (4)
O1—C8—C9	119.8 (3)	C5—C4—C9	118.8 (3)
C7—C8—C9	117.4 (3)	C2—C3—C4	120.2 (4)
C8—C7—C6	121.4 (4)	C2—C3—H3	119.9
C8—C7—H7	119.3	C4—C3—H3	119.9
C6—C7—H7	119.3	N2—C10—C11	123.1 (4)
N1—C9—C8	119.5 (3)	N2—C10—H10	118.5
N1—C9—C4	119.8 (3)	C11—C10—H10	118.5
C8—C9—C4	120.6 (3)	N1—C1—C2	123.6 (4)
C12—C13—C18	123.1 (3)	N1—C1—H1	118.2
C12—C13—C14	117.3 (4)	C2—C1—H1	118.2
C18—C13—C14	119.7 (3)	C11—C12—C13	120.5 (4)
O2—C15—C16	122.3 (3)	C11—C12—H12	119.7
O2—C15—C14	118.9 (3)	C13—C12—H12	119.7
C16—C15—C14	118.8 (3)	C6—C5—C4	120.0 (4)
C1—N1—C9	119.2 (3)	C6—C5—H5	120.0
C1—N1—Ag1	119.6 (3)	C4—C5—H5	120.0
			
C16—C17—C18—C13	−2.2 (7)	Ag1—N2—C14—C13	165.2 (2)
N2—Ag1—O1—C8	−173.2 (2)	C10—N2—C14—C15	177.8 (3)
N1—Ag1—O1—C8	−10.6 (2)	Ag1—N2—C14—C15	−14.3 (4)
Ag1—O1—C8—C7	−172.3 (3)	C12—C13—C14—N2	1.3 (5)
Ag1—O1—C8—C9	9.9 (4)	C18—C13—C14—N2	−178.9 (3)
O1—C8—C7—C6	−179.3 (4)	C12—C13—C14—C15	−179.2 (3)
C9—C8—C7—C6	−1.4 (5)	C18—C13—C14—C15	0.6 (5)
C5—C6—C7—C8	1.1 (6)	O2—C15—C14—N2	−1.4 (5)
O1—C8—C9—N1	−2.1 (5)	C16—C15—C14—N2	178.7 (3)
C7—C8—C9—N1	−180.0 (3)	O2—C15—C14—C13	179.1 (3)
O1—C8—C9—C4	178.4 (3)	C16—C15—C14—C13	−0.9 (5)
C7—C8—C9—C4	0.5 (5)	N1—C9—C4—C3	1.9 (5)
C17—C18—C13—C12	−179.3 (4)	C8—C9—C4—C3	−178.6 (3)
C17—C18—C13—C14	0.9 (6)	N1—C9—C4—C5	−178.8 (3)
C8—C9—N1—C1	177.5 (3)	C8—C9—C4—C5	0.7 (5)
C4—C9—N1—C1	−3.0 (5)	C1—C2—C3—C4	−1.6 (6)
C8—C9—N1—Ag1	−9.7 (4)	C5—C4—C3—C2	−178.9 (4)
C4—C9—N1—Ag1	169.8 (2)	C9—C4—C3—C2	0.4 (6)
N2—Ag1—N1—C1	−57.9 (5)	C14—N2—C10—C11	1.4 (6)
O1—Ag1—N1—C1	−176.8 (3)	Ag1—N2—C10—C11	−167.0 (3)
N2—Ag1—N1—C9	129.4 (4)	C12—C11—C10—N2	1.3 (6)
O1—Ag1—N1—C9	10.4 (2)	C9—N1—C1—C2	1.8 (6)
N1—Ag1—N2—C10	−84.3 (4)	Ag1—N1—C1—C2	−171.1 (3)
O1—Ag1—N2—C10	27.5 (3)	C3—C2—C1—N1	0.5 (7)
N1—Ag1—N2—C14	107.7 (4)	C10—C11—C12—C13	−2.7 (6)
O1—Ag1—N2—C14	−140.4 (2)	C18—C13—C12—C11	−178.3 (4)
O2—C15—C16—C17	179.7 (4)	C14—C13—C12—C11	1.5 (6)
C14—C15—C16—C17	−0.4 (6)	C7—C6—C5—C4	0.2 (6)
C18—C17—C16—C15	2.0 (7)	C3—C4—C5—C6	178.2 (4)
C10—N2—C14—C13	−2.7 (5)	C9—C4—C5—C6	−1.1 (6)

### Hydrogen-bond geometry (Å, º)

**Table:**

*D*—H···*A*	*D*—H	H···*A*	*D*···*A*	*D*—H···*A*
O2—H2*A*···O1^i^	0.82	1.73	2.495 (3)	154

Symmetry code: (i) *x*−1, *y*, *z*.
